# Bivariate longitudinal data analysis: a case of hypertensive patients at Felege Hiwot Referral Hospital, Bahir Dar, Ethiopia

**DOI:** 10.1186/s13104-017-3044-4

**Published:** 2017-12-08

**Authors:** Demeke Lakew Workie, Dereje Tesfaye Zike, Haile Mekonnen Fenta

**Affiliations:** Department of Statistics, Bahir Dar University, Peda Campus, P.O.Box: 79, Bahir Dar, Ethiopia

**Keywords:** Joint mixed effect models, Systolic blood pressure, Diastolic blood pressure hypertension

## Abstract

**Objective:**

Longitudinal data are often collected to study the evolution of biomedical markers. The study of the joint evolution of response variables concerning hypertension over time was the aim of this paper. A hospital based retrospective data were collected from September 2014 to August 2015 to identify factors that affect hypertensive. The joint mixed effect model with unstructured covariance was fitted. A total of 172 patients screened for antihypertensive drugs treated were longitudinally considered from Felege Hiwot referral.

**Results:**

The joint mixed effect model with unstructured covariance (AIC: 12,236.9 with $\chi_{12}^{2}$ = 1007.8, P < 10^−4^) was significantly best fit to the data. The correlation between the evolutions of DBP and SBP was 0.429 and the evolution of the association between responses over-time was found 0.257. Among all covariates included in joint-mixed-effect-models, sex, residence, related disease and time were statistically significant on evolution of systolic and diastolic blood pressure. The joint modeling of longitudinal bivariate responses is necessary to explore the association between paired response variables like systolic and diastolic blood pressure. Fitting joint model with modern computing method is recommended to address questions for association of the evolutions with better accuracy.

## Introduction

Hypertension is a chronic condition resulting from high blood pressure in the arteries during circulation. Clinically, a person is said to be hypertensive if the individual’s systolic blood pressure (SBP) is greater than 140 mm Hg and or diastolic blood pressure (DBP) is greater than 90 mmHg and those who were already under medication [1, 2]. Monitoring hypertension is a necessity for patients to assess the progression of blood pressure. Typically two markers (systolic and diastolic blood pressure, SBP and DBP) are measured repeatedly over time after treatment to ensure that no signs of blood pressure problems. These markers are needed to ensure an accurate evaluation of the blood pressure since they are correlated and could be influenced by the socioeconomic status of the patient [3]. Given the interdependence of these outcomes in determining blood pressure, it is important to evaluate the factors that affect the rate of change in these outcomes in a joint manner [4].

When several markers are measured repeatedly, joint modeling was used in the context of jointly studying time to clinical event and repeated measures on surrogate outcomes [5–8]. Others included joint modeling of the multilevel item response theory (MLIRT) and Cox’s proportional hazard model for time to dependent terminal event with shared random effects to link the two models [9, 10]. As Jaffa et al. [5], Thiebaut et al. [11] and Fiewus and Verebeke [12–14] stated a joint modeling of biomarker data is necessary to quantify, the relationship between evolutions of different responses and the evolution of the relationship between different response variables over time. Thus, the aim of this study was using the joint mixed effect model with unstructured covariance structure to answer the evolution of systolic and diastolic blood pressure over time.

## Main text

### Methods

Hospital based retrospective studies were conducted among hypertensive patients attending antihypertensive clinic between September 2014 to August 2015 at Felege Hiwot referral hospital, Bahir Dar, Ethiopia. The area is selected because it is central referral hospital that provides organized hypertension follows up care. The sample size was calculated using 95% confidence level and the overall sample size was 172 which after adding 5% for non-response. A systematic random sampling method was adopted for selecting a representative sample from the list of the medical charts that contain the list of hypertensive patients’ name and identification number. Patients were selected randomly using their unique identification number. The study considered all hypertensive patients, whose age was above 15 years regardless of their treatment category during the study period in the referral hospital.

In this paper, a longitudinal data on two markers of hypertension (SBP and DBP), various socioeconomic status covariates and clinical factors were considered. SBP and DBP measurements were collected at baseline (SBP0 and DBP0), and every 3 months (SBP3 to SBP12 and DBP3 to DBP12) thereafter. In order to ensure sufficient available information, only a sub-sample of patients having at least four repeated with equally spaced measurements were included in the analyses. Thus, a follow-up data were included hypertensive patients had a maximum of five repeated measures on blood pressure. Data were entered and edited in SPSS v. 21 and analyzed by SAS v. 9.4 and the 95% confidence interval was used to determine significance tests. The missing data for both markers were replaced by last observation carried forward (LOCF) as [15].

#### Bivariate random mixed effect model

Multivariate longitudinal data arise when a set of different responses on the same unit are measured repeatedly over time. A joint modeling of such kind of data is necessary to quantify, firstly, the relationship between evolutions of different responses and, secondly, the evolution of the relationship between different response variables over time. Thus, a pair wise fitting approach has been used in this study as proposed in the literature [12, 16]. The joint modeling approach investigated in this study was the bivariate longitudinal mixed effect models that included both fixed and random effects. Thus, in this study DBP_ij1_ and SBP_ij2_ be the bivariate outcomes for the ith subject measured at jth times for outcomes 1 and 2.

We define a general bivariate linear mixed effect model including a random component, a variance–covariance process, and an independent error. Let $$ Y^{k}_{i} = \left[ {\begin{array}{*{20}c} {DBP_{i} } \\ {SBP_{i} } \\ \end{array} } \right] $$, the response vector for the ith subject with $DBP_{i}$ and $SBP_{i}$ having n_i_ sampled measurements of the marker k (k = 1, 2).

To take into account correlation between both markers the following bivariate linear mixed effect model was used.

The mixed-effect models assume that the regression coefficients are a random sample from some population of the possible coefficient and allow one to model variations between study units [17]. The random Coefficient mixed model gives that two random slopes (one for DBP and one for SBP) be fitted for each individual and the variances of measurement errors are different for different markers. The covariance matrix for the random slopes is $$ G_{{}} = \left[ {\begin{array}{*{20}c} {\sigma_{1}^{2} } & {\sigma_{12}^{2} } \\ {\sigma_{21}^{2} } & {\sigma_{2}^{2} } \\ \end{array} } \right] $$, and the two markers are independent of $$ G_{{}} = \left[ {\begin{array}{*{20}c} {\sigma_{1}^{2} } & 0\\ 0& {\sigma_{2}^{2} } \\ \end{array} } \right] $$.

In this paper, a bivariate linear mixed model including random effects and independent measurement error for both SBP and DBP was presented as outcome variables in each occasion. The two end points were longitudinally measured as a vector of responses, Y_i_, at each occasion with this model: $Y_{i} = X_{i} \beta + Z_{i} \gamma_{i} + W_{i} + \varepsilon_{i}$ with $$ \left\{ {\begin{array}{*{20}c} {\varepsilon_{i}^{{}} \sim N(0,\varSigma_{i}^{{}} )} \\ {W_{i} \sim N(0,R_{i} )} \\ {\gamma_{i}^{{}} \sim N(0,G)} \\ \end{array} } \right. $$, where $$ X_{i} = \left[ {\begin{array}{*{20}c} {X_{i}^{1} } & 0 \\ 0 & {X_{i}^{2} } \\ \end{array} } \right] $$, $$ \beta = \left[ {\begin{array}{*{20}c} {\beta_{1} } \\ {\beta_{2} } \\ \end{array} } \right] $$, $$ Z_{i} = \left[ {\begin{array}{*{20}c} {Z_{i}^{1} } & 0 \\ 0 & {Z_{i}^{2} } \\ \end{array} } \right] $$, $$ \gamma_{i} = \left[ {\begin{array}{*{20}c} {\gamma_{i}^{1} } \\ {\gamma_{i}^{2} } \\ \end{array} } \right] $$ and $$ W_{i} = \left[ {\begin{array}{*{20}c} {W_{i}^{1} } \\ {W_{i}^{2} } \\ \end{array} } \right] $$ is a $2n_{i}$-vector of realization of a variance–covariance process $$ w_{i} (t) = \left[ {\begin{array}{*{20}c} {w_{i}^{1} (t)} \\ {w_{i}^{2} (t)} \\ \end{array} } \right] $$ and $$ \varepsilon_{i} = \left[ {\begin{array}{*{20}c} {\varepsilon_{i}^{1} } \\ {\varepsilon_{i}^{2} } \\ \end{array} } \right] $$ represents independent measurement errors. Though many socioeconomic covariates and clinical factors were considered in the analysis, only covariates significantly associated with systolic and diastolic blood pressure were reported.

### Results

The sample was composed of 172 patients with antihypertensive drugs treated, of which 38 (22.09%) and 21 (12.51%) patients had diabetic and stroke and 76 (44.19%) patients had no other related disease. The results showed that, 93 (54.09%) women, with a mean age of 51.87 (SD = 14.33) years. Whereas mean age of male patients was 56.82 (SD = 14.98) years and the mean systolic and diastolic blood pressure of patients declined from baseline to the next 3 months period, to the next 6 months period and soon up to the last 12 months period follow up time. The baseline SBP mean of patients was 153.58 (SD = 31.60) mmHg and declined to 129.48 (SD = 20.07) mmHg over time and similar history was found in DBO patients. These summaries suggest that the treatment has a significant effect on systolic and diastolic blood pressure over the follow-up time (Table 1).

**Table 1:** Patients’ demographic characteristics

Variables	n (%)	Mean (SD)	Min–max	95% CI
Sex				
Male	79 (45.93)			
Female	93 (54.07)			
Residence				
Urban	101 (59.06)			
Rural	70 (40.94)			
Other related disease				
None	76 (44.19)			
Others	37 (21.51)			
Stroke	21 (12.51)			
Diabetic	38 (22.09)			
Age in years				
Male	79	56.82 (14.49)	20.00–86.00	53.58–60.07
Female	92	51.87 (14.33)	25.00–92.00	48.90–54.84
Total	171	54.16 (14.57)	20.00–92.00	51.96–56.36
SBP in mmHg				
SBP0	172	153.58 (31.60)	60.00–240.00	148.82–158.33
SBP1	172	142.47 (23.33)	80.00–210.00	138.96–145.98
SBP2	172	135.78 (22.99)	80.00–220.00	132.33–139.24
SBP3	172	133.84 (23.18)	80.00–240.00	130.35–137.33
SBP4	172	129.48 (20.07)	80.00–200.00	126.46–132.50
DBP in mmHg				
DBP0	172	94.22 (17.80)	50.00–160.00	91.54–96.89
DBP1	172	86.54 (15.30)	40.00–140.00	84.24–88.84
DBP2	172	82.96 (14.36)	20.00–140.00	80.80–85.12
DBP3	172	82.67 (13.33)	50.00–130.00	80.67–84.68
DBP4	172	83.02 (13.25)	50.00–170.00	81.03–85.02

*SD* standard deviation, *Min–Max* minimum–maximum, *CI* confidence interval, *SBP* systolic blood pressure, *DBP* diastolic blood pressure, *mmHg* millimeter mercury

**Fig. 1:**
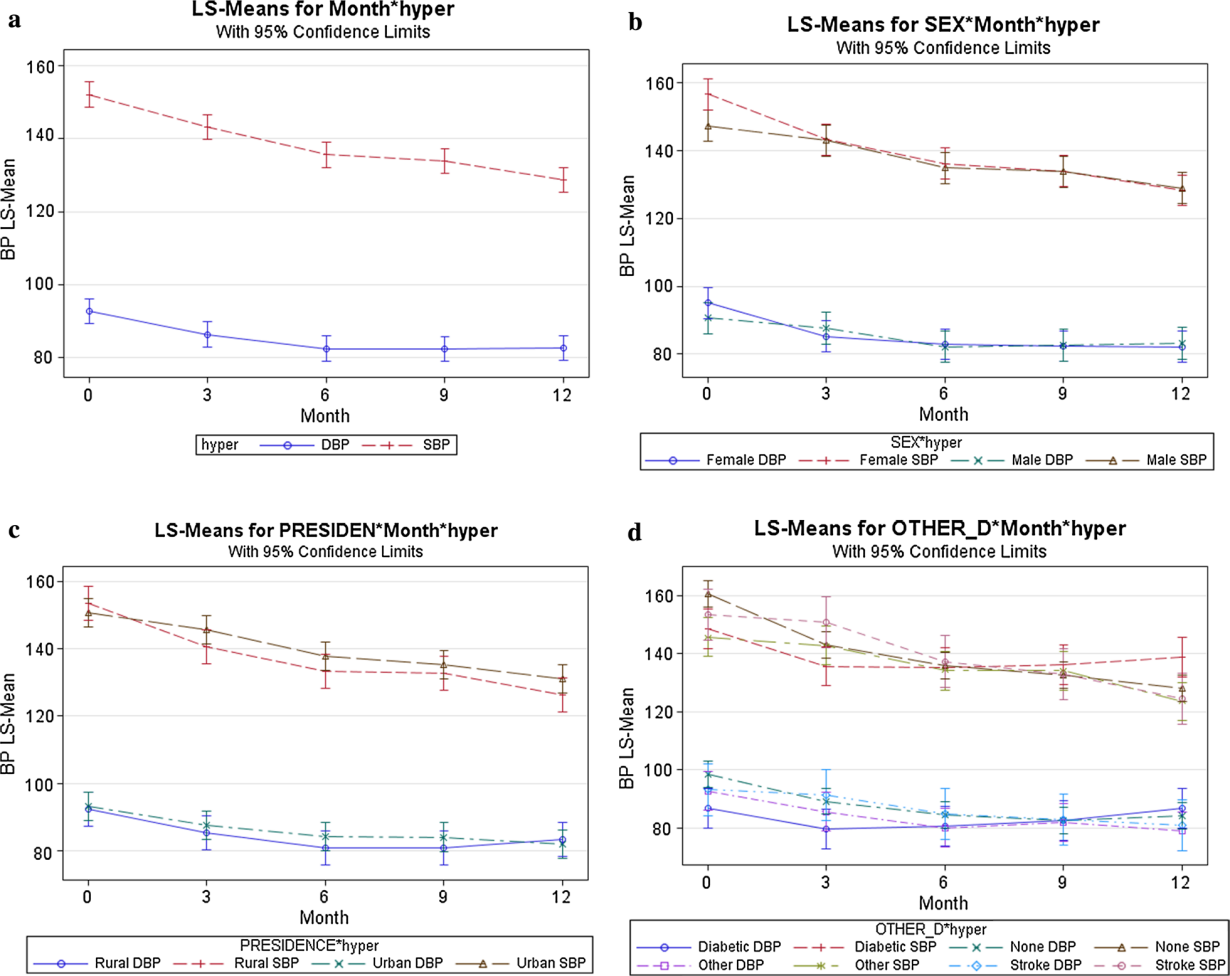
Mean estimates with 95% CI **a** Systolic and diastolic BP. **b** Systolic and diastolic BP by sex. **c** Systolic and diastolic BP by place of residence. **d** Systolic and diastolic BP by related disease

Figure 1 shows mean estimates with 95% CI, for the SBP and DBP separately by different factors. Clearly, observe that decreasing trend in both SBP and DBP over time. The mean estimate of blood pressure for a female was more than male at baseline and significantly different (P  < 0.0001) between the outcomes. Whereas, the mean estimate of diastolic and systolic blood pressure for rural and urban area seems to no difference The bivariate random mixed-effects model was fitted to show the association of correlate measurements to the 3-month-interval for blood pressure to identify the risk factors.

The bivariate random mixed-effects model was significantly best with AIC value of 12,236.9 and $\chi_{12}^{2}$ = 1007.8, P < 10^−4^. Output obtained by the bivariate random mixed effects model with UN provides estimations for the covariance matrix and fixed effects. The variance–covariance matrix for the random effects and the error components for each response were obtained as:

$$ \begin{aligned} \text{G} & \text{ = }\left[ {\begin{array}{*{20}c} {\sigma_{{a_{1} }}^{2} } & {\sigma_{{a_{1} b_{1} }} } & {\sigma_{{a_{1} a_{2} }} } & {\sigma_{{a_{1} b_{2} }} } \\ {} & {\sigma_{{b_{1} }}^{2} } & {\sigma_{{b_{1} a_{2} }} } & {\sigma_{{b_{1} b_{2} }} } \\ {} & {} & {\sigma_{{a_{2} }}^{2} } & {\sigma_{{a_{2} b_{2} }} } \\ {} & {} & {} & {\sigma_{{b_{2} }}^{2} } \\ \end{array} } \right] = \left[ {\begin{array}{*{20}c} {153.21} & {169.80} & {76.13} & {168.90} \\ {} & {402.77} & {116.83} & {435.72} \\ {} & {} & {219.62} & {171.44} \\ {} & {} & {} & {725.77} \\ \end{array} } \right]\;\text{and} \\ \text{R} & = \left[ {\begin{array}{*{20}c} {\sigma_{1}^{2}       \sigma_{12} } \\ {             \sigma_{2}^{2} } \\ \end{array} } \right] = \left[ {\begin{array}{*{20}c} {58.76} & {90.61} \\ {} & {194.55} \\ \end{array} } \right] \\ \end{aligned} $$

The correlation between the evolutions for the two random slopes is given by:

$$ \text{r}_{\text{E}} = \frac{{Cov\left( {b_{1} ,b_{2} } \right)}}{{\sqrt {Var\left( {b_{1} } \right)*Var\left( {b_{2} } \right)} }} = \frac{{\sigma_{{b_{1} b_{2} }} }}{{\sqrt {\sigma_{{b_{1} }}^{2} *\sigma_{{b_{2} }}^{2} } }} = \frac{171.44}{{\sqrt {219.62*725.77} }} = 0. 4 2 9 $$

whereas, the marginal correlation between the two responses as a function of time is given by:

$$ \begin{aligned} \text{r}_{\text{m}} (\text{t})  = \frac{{\sigma_{{a_{1} a_{2} }} + t\left( {\sigma_{{a_{1} b_{2} }} + \sigma_{{b_{1} a_{2} }} } \right) + t^{2} \sigma_{{b_{1} b_{2} }} }}{{\sqrt {\left( {\sigma_{{a_{1} }}^{2} + 2t^{2} \left( {\sigma_{{a_{1} b_{1} }} + \sigma_{{b_{1} }}^{2} } \right) + \sigma_{1}^{2} } \right)*\left( {\sigma_{{a_{2} }}^{2} + 2t^{2} (\sigma_{{a_{2} b_{2} }} + \sigma_{{b_{2} }}^{2} } ) + \sigma_{2}^{2}\right )} }} \\  \quad \times \frac{{76.13 + t\left( {168.9 + 116.83} \right) + t^{2} 435.72}}{{\sqrt {\left( {153.21 + 2t^{2} \left[ {169.8 + 402.77} \right] + 58.76} \right)*\left( {219.62 + 2t^{2} \left[ {171.44 + 725.77} \right] + 194.55} \right)} }} \\  = \frac{{435.72t^{2} + 285.73t + 76.13}}{{\sqrt {\left( {211.97 + 1145.4t^{2}  } \right)*\left( {414.17 + 1794.42t^{2} } \right)} }} = \left[ {\begin{array}{*{20}c} {0.257} \\ {0.368} \\ {0.337} \\ {0.327} \\ {0.320} \\ \end{array} } \right] \\ \end{aligned} $$

The result revealed that the correlation between the diastolic and systolic blood pressure for random intercepts at baseline was 0.257 and the remaining marginal correlation for 3 to 12 months were 0.368, 0.337, 0.327 and 0.320, respectively over the 3 month period follow up time.

The primary focus of this study was interested in modeling longitudinal measures of SBP and DBP patients as a function of baseline age, sex, residence and related disease. The bivariate random mixed-effects model revealed that baseline (P  = 0.0001), 3 months period (P  = 0.001) and 6 months period (P  = 0.011) systolic and diastolic blood pressure were significantly different compared with 12 months period follow up time. The systolic and diastolic blood pressure of rural patients (P  = 0.0308) was significantly different compared with urban patients. The systolic and diastolic blood pressure patients with diabetic showed a significant difference at baseline (P  = 0.0077) and 3 months period (P  = 0.0015) compared to patients with stroke (Table 2).

**Table 2:** Solution for fixed effects

Effect	Category	Estimate	Standard error	DF	t value	Pr > |t|
Age		− 0.07951	0.04934	1341	− 1.61	0.1073
Sex (male = ref)	Female	130.26	4.6367	1341	28.09	*<* *.0001*
Residence (urban = ref)	Rural	2.6875	2.2787	1341	1.18	0.2385
Other disease (stroke = ref)	Diabetic	4.4679	4.0411	1341	1.11	0.2691
	None	2.4592	3.6329	1341	0.68	0.4986
	Other	− 1.8215	4.0377	1341	− 0.45	0.6520
Month (after 12 months = ref)	0 = Baseline	25.4167	4.7397	1341	5.36	*<* *.0001*
	3 = After 3 months	22.7743	4.7397	1341	4.81	*<* *.0001*
	6 = After 6 months	12.0668	4.7397	1341	2.55	*0.0110*
	9 = After 9 months	8.2842	4.7397	1341	1.75	0.0807
Sex*month (male*12 = ref)	Female*0	3.7734	2.7517	1341	1.37	0.1705
	Female*3	− 1.9921	2.7517	1341	− 0.72	0.4692
	Female*6	0.8489	2.7517	1341	0.31	0.7577
	Female*9	0.1634	2.7517	1341	0.06	0.9527
Residence*month (urban*12 = ref)	Rural*0	− 3.5497	2.8104	1341	− 1.26	0.2068
	Rural*3	− 4.3474	2.8104	1341	− 1.55	0.1221
	Rural*6	− 6.0771	2.8104	1341	− 2.16	*0.0308*
	Rural*9	− 5.0831	2.8104	1341	− 1.81	0.0707
Other disease*month (stroke*12 = ref)	Diabetic*0	− 13.2490	4.9672	1341	− 2.67	*0.0077*
	Diabetic*3	− 17.3071	4.9672	1341	− 3.48	*0.0005*
	Diabetic*6	− 9.8464	4.9672	1341	− 1.98	0.0477
	Diabetic*9	− 5.5524	4.9672	1341	− 1.12	0.2638
	None*0	1.1267	4.4649	1341	0.25	0.8008
	None*3	− 4.9565	4.4649	1341	− 1.11	0.2672
	None*6	− 3.0551	4.4649	1341	− 0.68	0.4939
	None*9	− 2.6169	4.4649	1341	− 0.59	0.5579
	Other*0	1.2464	4.9616	1341	0.25	0.8017
	Other*3	− 4.0513	4.9616	1341	− 0.82	0.4143
	Other*6	− 2.7634	4.9616	1341	− 0.56	0.5777
	Other*9	0.3122	4.9616	1341	0.06	0.9498

*DF* degree of freedom, *ref* reference category

### Discussion

The joint mixed effect model with unstructured covariance (AIC 12,236.9 with $\chi_{12}^{2}$ = 1007.8, P < 10^−4^) was significantly best fit to the covariates. This results in line with [18, 19] as they used a joint model for a longitudinal data and obtained the best final model with UN compared to others.

The correlation between the evolutions for the DBP and SBP was 0.429. This result was in line with a joint model fitted for female SBP and DBP that considered the before and after drug administration data [20]. Similarly, the evolution of the association between the responses over time was found 0.257. The remaining marginal correlation between DBP and SBP was 0.368, 0.337, 0.327 and 0.320, respectively over the follow-up time. As the marginal correlation converges to the random slopes, the relationship was underlined by the correlation between DBP and SBP at each follow-up time [12].

The mean difference in systolic and diastolic blood pressure for male and female had only significant (P  < 0.034) between the baseline and after a 12-month follow-up-time. This result in line with [21–23] as they stated hypertension is the leading cause of death in women than that of men and they concluded that antihypertensive treatment decreases the death of women. The current study revealed that there is an association between blood pressure at baseline and during follow-up among patients with stroke and diabetes. This result was similar to the study in the Louisiana State University Hospital-based longitudinal data [24–26], and blood pressure at baseline and during follow-up and the risk of all-cause mortality among patients with diabetes [27].

### Conclusion and recommendation

The joint mixed effect model with unstructured covariance was preferred among others to fit the data. It can be generalized that, the two outcomes have a strong positive correlation and the joint mixed effect model was preferred. Thus, joint modeling of longitudinal bivariate responses is necessary to explore the association between paired response variables. The baseline mean of the two symptoms was out of the normal range for hypertensive patients but it declines through clinical treatment follow-up time of 3 month period intervals. As the joint model is able to address questions for the association of the evolutions with better accuracy, fitting joint mixed effect model is recommended.

## Limitations

The joint modeling problem is failing to convergence because of a large number of parameter estimates. Moreover, some one also might want to look at modeling more than two response variables over time. Thus one might be able to be implemented using modern computing method for future work to model more than two response variables over time. Here in the joint mixed effect model, authors did not see the interaction effect of the predictors over time. Therefore, researchers should consider the contribution of the interaction effect of the predictors on to the joint mixed effect model.

## Abbreviations

AIC: Akaike Information Criterion
CI: confidence interval
DBP: diastolic blood pressure
LOCF: last observation carried forward
MLIRT: multilevel item response theory
SAS: statistical analysis system
SBP: systolic blood pressure
SD: standard deviation
SPSS: Statistical package for Social Science
UN: unstructured
